# A Severe Pharyngeal-Sensory-Ataxic Variant of Guillain-Barré Syndrome With Transient Cardiac Dysfunction and a Positive Anti-sulfatide IgM

**DOI:** 10.7759/cureus.29261

**Published:** 2022-09-17

**Authors:** King Peng Lee, Sanihah Abdul Halim, Nur Asma Sapiai

**Affiliations:** 1 Department of Neurosciences, School of Medical Sciences, Universiti Sains Malaysia, Kelantan, MYS; 2 Department of Internal Medicine/Neurology, School of Medical Sciences, Universiti Sains Malaysia, Kelantan, MYS; 3 Brain and Behaviour Cluster, School of Medical Sciences, Hospital Universiti Sains Malaysia, Kelantan, MYS; 4 Department of Radiology, School of Medical Sciences, Universiti Sains Malaysia, Kelantan, MYS

**Keywords:** guillain-barré syndrome, peripheral neuropathy, ganglioside antibody, sensory-ataxic variant, acute bulbar palsy

## Abstract

Guillain-Barré syndrome (GBS) is a heterogeneous group of acute immune-mediated polyradiculoneuropathy that typically presents with classic axonal or demyelinating sensory-motor type. However, there are variants of GBS with atypical presentation. We report a rare case of severe pharyngeal-sensory-ataxic variant of GBS associated with poor cardiac systolic function, elevated troponin, and positive anti-sulfatide IgM. The sensory symptom atypically started in the hands in an ascending pattern, which progressed to involve the trunk and face and, later, all limbs. It was associated with severe dysphagia, ataxia, and generalized areflexia but with preserved muscle strength in all extremities. Recognizing the atypical pattern of presentation and the ability to perform an accurate clinical localization are the utmost important initial steps in making the diagnosis. The patient showed complete recovery after immunoglobulin therapy.

## Introduction

Guillain-Barré syndrome (GBS) can present with various atypical presentations such as Miller Fisher syndrome (MFS), Bickerstaff’s brain stem encephalitis, pharyngeal-cervical-brachial motor, acute bulbar palsy, paraparetic motor, bilateral facial palsy with paresthesia, pure sensory, sensory-ataxic, and pan-dysautonomia variants. Some patients may present with overlapping variants, associated with different forms of anti-ganglioside antibodies. Cardiac involvement is rare, but it has been reported. The history of antecedent infection provides a clue to the diagnosis. Our patient presented with a unique pattern of a severe variant of GBS, and the only positive immunological evidence found was the anti-sulfatide IgM antibody. Whether this is a new entity of GBS or is part of a previously described overlapping variant requires further study.

## Case presentation

A 23-year-old male, with no significant medical history, presented with progressive numbness starting from bilateral fingers ascending to the proximal upper limbs. The symptoms spread to the chest, neck, face, and abdomen and finally involved the distal lower limbs in an ascending manner up to the thigh region over a period of one week. Within the same week, he experienced progressive severe dysphagia associated with nasal fluid regurgitation and nasal speech. Two days prior to admission, he developed unsteadiness of gait. He had an antecedent throat infection one month prior, which had resolved.

Otherwise, there was no history of fever, headache, vertigo, vomiting, or limb weakness. There were no autonomic or cardiac symptoms, no risk factors for coronary artery disease, and no symptoms of connective tissue disease. There was no family history of neurological disorders or sudden cardiac death. There was no history of recent illicit drug use, alcohol abuse, or toxin exposure.

On examination, he had normal vital signs and was afebrile. There was bulbar palsy with reduced palatal movements predominantly on the right side as well as weak gag reflexes bilaterally, pooling of saliva, and hoarseness of voice with a nasal tone. The facial sensation to pinprick and light touch was impaired bilaterally, but the other cranial nerves were intact. Muscle strength was normal in all limbs but with generalized hyporeflexia, which later became areflexic. Impairment of all sensory modalities (pinprick, light touch, temperature, vibration, and proprioception) was found in all limbs, trunk, and neck. There was also evidence of bilateral dysmetria and ataxic gait. The Romberg test was positive. Other systemic examinations were unremarkable. He deteriorated on the second day of admission with respiratory failure due to aspiration pneumonia as a result of bulbar palsy. He was subsequently intubated.

**Figure 1:**
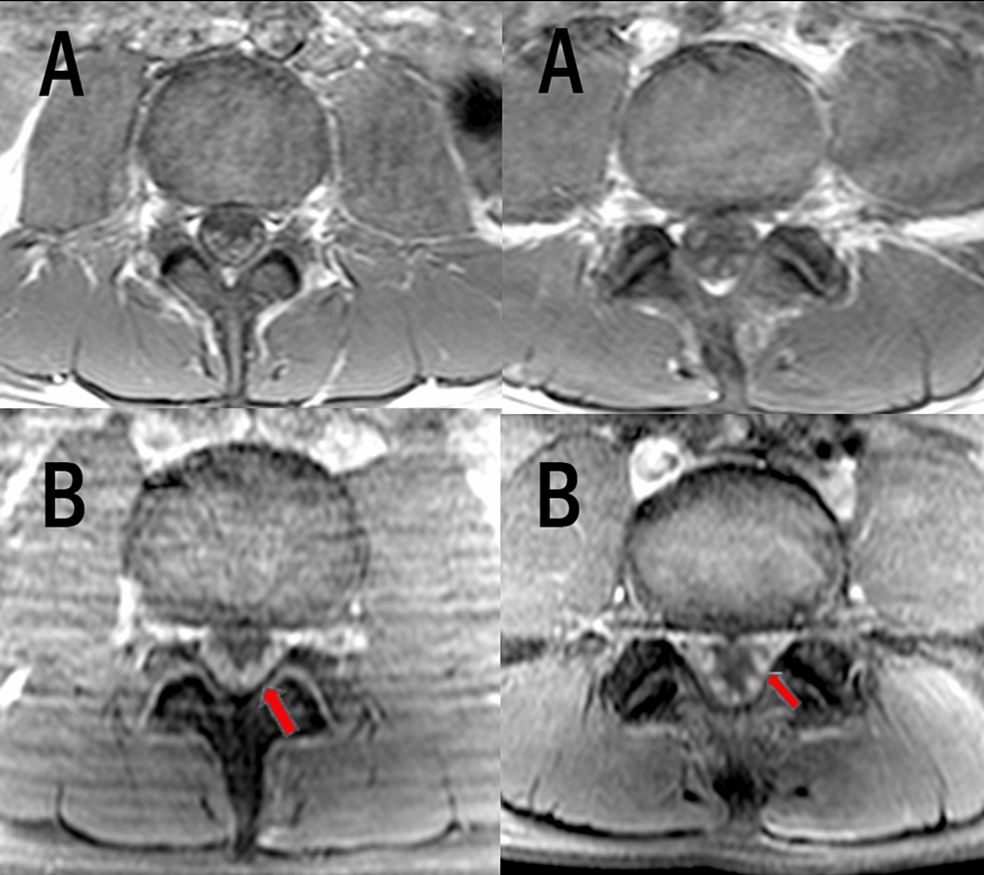
A and B show two levels of cauda equina. A (spine MRI axial T1 pre-contrast) and B (spine MRI axial T1 post-contrast) show contrast enhancement of cauda equina nerve roots (arrows). MRI: magnetic resonance imaging

Baseline blood investigations were normal. A nerve conduction study (NCS) was immediately performed (on day 7 of illness) and revealed a normal sensory study with a mild reduction of compound muscle action potential (CMAP) amplitude over the right median nerve. Contrast-enhanced brain magnetic resonance imaging (MRI) was unremarkable. Whole-spine MRI was performed, which showed cauda equina nerve root enhancement (Figure 1) with a normal spinal cord.

Electrocardiography (ECG) on admission showed ST depression and T inversion over inferior and anterolateral leads. Troponin T was also significantly elevated at 257 ng/L (reference value: <14 ng/L). There was no cardiac symptom, but he had mild tachycardia with normal blood pressure. Serial troponin tests showed further elevation up to 799 ng/L. Echocardiography revealed a left ventricular ejection fraction of 40%-45% with global hypokinesia. The cardiac abnormalities may be contributed by dysautonomia.

Lumbar puncture revealed features of albuminocytologic dissociation with acellular and elevated protein levels of 1.98 g/L in the cerebrospinal fluid with a normal glucose level.

Ganglioside autoimmune antibodies were measured on day 11 of illness and included both IgM and IgG antibody tests for sulfatide (GM1, GM2, GM3, GM4, GD1a, GD1b, GD2, GD3, GT1a, GT1b, and Gq1b). The result showed a borderline titer for anti-sulfatide IgM and anti-GM4 IgM. In view of the borderline titer of the ganglioside antibody results, the tests were repeated four weeks after the first samples, which showed a positive sulfatide IgM with a borderline titer of GM4 IgM.

COVID-19 polymerase chain reaction was negative. Other blood tests were normal: connective tissue and vasculitis screening, viral and mycoplasma screening, erythrocyte sedimentation rate, blood cultures, thyroid function test, and paraproteinemia screening. Urine drug toxicology and urine cultures were both negative.

He was started on a course of intravenous immunoglobulin 0.4 g/kg daily for five days. His hospitalization was complicated by a right pneumothorax due to barotrauma, massive blood loss with hypotension, and disseminated intravascular coagulation secondary to vascular injury during chest tube insertion as well as nosocomial pneumonia. He underwent physiotherapy in the intensive care unit.

After one month of treatment, his general condition improved. He was weaned off ventilation and was extubated well. Neurological reassessment a month after immunoglobulin revealed improvement of fine touch sensation over the face and upper chest as well as the bulbar function. However, sensory deficits were still present in all limbs and the abdominal region with hyporeflexia and residual ataxia. A repeat NCS after a month in intensive care showed the absence of median sensory nerve action potential (SNAP) bilaterally with moderate reduction of SNAP amplitude over bilateral ulnar nerves but an intact bilateral sural SNAP. Motor NCS showed a diffuse moderate reduction of CMAP amplitude with a normal conduction velocity. Based on the NCS, there was a non-length-dependent type of sensory neuropathy that predominantly involved the upper limbs and a subclinical axonal type of motor neuropathy.

Echocardiography was repeated and showed normalization of myocardial contractility without any hypokinetic segments. Two months after the onset, the patient was able to walk without support but unable to perform tandem gait. Dysphagia resolved completely with residual hyperesthesia of both hands and feet. Three months later, his symptoms disappeared completely with a normal neurological examination.

## Discussion

Our patient presented with predominant pharyngeal weakness and diffuse sensory disturbance with ataxia, which is a nonclassical combination of manifestations in Guillain-Barré syndrome (GBS). It is crucial to exclude other diagnoses in a patient who presents with atypical features. In our case, normal imaging ruled out brainstem and intramedullary spinal cord lesions. Acute neuromuscular junction disorders such as myasthenic crisis or acquired myopathies can be excluded due to the presence of sensory symptoms.

The clinical diagnosis of GBS, in this case, was made based on a few important basic features: acute onset of ascending symmetrical numbness over bilateral upper and lower extremities, generalized areflexia, rapid progression within days, and antecedent infection [1]. The diagnosis was supported by investigations that included cerebrospinal fluid albuminocytologic dissociation, axonal neuropathy on electrodiagnostic tests, and a positive ganglioside antibody anti-sulfatide IgM.

In our case, the findings of diffuse sensory disturbance that atypically started in the bilateral hands as well as severe bulbar palsy, ataxia, and cardiac dysfunction suggest a rare variant of GBS, unlike the classical sensory-motor form that usually starts in the lower extremities in an ascending pattern [1]. Dysphagia in this case is due to motor impairment rather than just an impaired throat sensation as evidenced by asymmetrical impairment of palatal movement during the examination. In GBS, severe acute bulbar palsy is usually notable in only a few localized variants such as pharyngeal-cervical-brachial (PCB) or acute bulbar palsy plus (ABPp) syndrome. The PCB variant of GBS accounts for only 2%-4% of GBS cases [2]. However, typical PCB syndrome presents with a pure motor form that causes weakness of the oropharynx, neck, and proximal upper limbs but spares the lower limb muscles and without sensory impairment [3]. Nagashima et al. analyzed 100 patients with PCB with overlapping variants [4]. The most frequent symptoms were arm weakness (29%), followed by dysphagia (17%), and then diplopia (17%), whereas frequent examination findings were hyporeflexia/areflexia, sensory impairment, external ophthalmoplegia, and ataxia due to overlapping presentations with Miller Fisher syndrome (MFS) and sensory-motor type of GBS [4,5].

Two case series reported a localized bulbar variant of GBS in an Asian population [6,7]. Kim et al. proposed a new subset of GBS variants with acute bulbar palsy in their case study of 11 Asian subjects [7]. Acute bulbar palsy plus (ABPp) syndrome is characterized by a prominent acute pharyngeal weakness in combination with other cranial symptoms or ataxia, without limb or neck weakness [6]. Cao et al. analyzed 28 Asian patients with ABPp syndrome [6]. The main features include acute onset of bulbar dysfunction in association with ocular weakness (85.7%), facial palsy (60.7%), or ataxia (50%). Most patients in their series had antecedent upper respiratory tract or gastrointestinal infections [6].

Between these two bulbar variants, ABPp syndrome appears more likely than PCB in our case due to the presence of bulbar palsy and ataxia without neck or limb weakness. However, our patient did not have facial palsy or ophthalmoplegia, which were common in ABPp variants. Neither PCB nor ABPp variants can completely fit into our patients’ clinical profiles. In previous ABPp reports, sensory impairment of the limbs was found in 50% of cases [6]. Another variant that can cause predominant sensory symptoms with gait imbalance includes the sensory-ataxic form of GBS. The sensory-ataxic variant is characterized by acute symmetrical sensory loss, absent tendon reflexes, normal muscle strength, and at least two pieces of evidence for nerve demyelination, with other usual features of GBS [8]. Ito et al. analyzed 54 patients with GBS with ataxic variants and acute sensory-ataxic neuropathy; 85% had sensory disturbances, 37% had mild limb weakness, 7% had blepharoptosis, 7% had facial palsy, 7% had bulbar palsy, and 4% had autonomic dysfunction [9].

In our case, the only positive immunological marker was the antibody to sulfatide IgM. The initial result was borderline but became positive on a repeat test. Sulfatide is a common glycolipid found in the spinal cord and peripheral nerve tissue. It is highly abundant in myelin [10]. Anti-sulfatide antibodies can be present in a variety of disorders, including GBS, chronic inflammatory demyelinating polyradiculoneuropathy, sensory and sensorimotor axonal neuropathy, multiple sclerosis, idiopathic thrombocytopenic purpura, autoimmune chronic active hepatitis, and diabetic neuropathy [11]. A study involving 25 patients with elevated sulfatide antibodies found that 98% of patients have peripheral neuropathy [11]. Most patients with positive sulfatide antibodies had predominantly pure sensory neuropathy rather than the sensorimotor type: 80% had a predominantly axonal neuropathy and 12% had a primary demyelinating neuropathy [11].

Anti-sulfatide antibodies were found in 65% of patients with GBS and in 15% of healthy controls [12]. This high frequency of positive anti-sulfatide antibodies might be relevant to the pathogenesis of GBS [12]. In another study, only one out of 21 cases of GBS had positive anti-sulfatide antibodies. This was associated with the most profound sensory loss, similar to our case [13]. The antibody titers were the highest in the acute phase of the disease and returned to the normal range within three weeks, suggesting that the antibodies were related to the disease [13]. However, another study found a contradictory result in which none of the GBS cases had positive anti-sulfatide antibodies [14].

There is limited literature on the prevalence of anti-sulfatide antibodies in PCB, ABPp, and sensory-ataxic variants of GBS. The most common antibody found in the PCB variant is antiGT1a IgG (51% of patients). These antibodies bind to oropharyngeal and cervicobrachial muscle innervations, which explain the disease manifestations [4]. Other antibodies such as mGM1, GM1b, GM3, GD1a, GD1b, GT1a, or GalNAc-GD1a are less common in patients with PCB [2,3,15]. In one study, positive anti-sulfatide IgM was reported in a young man with a PCB variant of GBS [16]. The anti-GT1a IgG and anti-GQ1b were the two most common antibodies found in the ABPp variant [6,7]. At the time of writing this manuscript, there is no literature reporting the prevalence of anti-sulfatide antibodies in ABPp variants of GBS.

Another interesting observation in our patient is transient cardiac systolic dysfunction with significantly elevated cardiac troponin that occurred during the peak of the acute illness and recovered immediately upon the resolution of the neurological symptoms. Neurogenic stunned myocardium and autonomic dysfunction have been proposed to be possible mechanisms based on a few published case reports [17-19]. In one study of 96 GBS cases, cardiovascular complications were reported in 54% of cases. Abnormal ECG data were found in 50% of cases, and raised troponin T levels occurred in 3.1% of cases. Other common findings include hypertension, tachycardia, bradycardia, raised pro-brain natriuretic peptide (BNP), and labile blood pressure or heart rate. Uncommon features include acute coronary syndrome, heart failure, and abnormal echocardiography [20].

Based on the previous case series, most patients with ABPp variants of GBS had a monophasic course of disease with good recovery after immunotherapy such as intravenous immunoglobulin or plasma exchange [6,7]. The majority of patients (89.3%) recovered completely within five months after the onset of illness [6]. In our case, the patient had a good recovery after five months of treatment.

## Conclusions

GBS can present with a severe form of overlap variant with atypical neurological presentation and cardiac dysfunction. Severe acute bulbar palsy is usually notable in a few localized GBS variants such as pharyngeal-cervical-brachial (PCB) or acute bulbar palsy plus (ABPp) syndrome. Recognizing the atypical pattern of presentation and the ability to perform an accurate clinical localization are the utmost important initial steps to reaching the diagnosis to provide prompt emergency treatment.
